# Challenges to implementation and strengthening of initial COVID-19 surveillance in Vanuatu: January–April 2020

**DOI:** 10.5365/wpsar.2020.11.2.012

**Published:** 2021-04-05

**Authors:** Wendy Williams, Caroline van Gemert, Joanne Mariasua, Edna Iavro, Debbie Fred, Johnny Nausien, Obed Manwo, Vincent Atua, George Junior Pakoa, Annie Taissets, Tessa B Knox, Michael Buttsworth, Geoff Clark, Matthew Cornish, Posikai Samuel Tapo, Len Tarivonda, Philippe Guyant, on behalf of the Vanuatu Ministry of Health’s National Health Emergency Operations Centre

**Affiliations:** a Department of Public Health, Vanuatu Ministry of Health, Port Vila, Vanuatu.; b Vanuatu Health Program, Port Vila, Vanuatu.; c Melbourne School of Population and Global Health, The University of Melbourne, Parkville, Victoria, Australia.; d Burnet Institute, Melbourne, Victoria, Australia.; e Vila Central Hospital, Port Vila, Vanuatu.; f Country Liaison Office, World Health Organization, Port Vila, Vanuatu.; g Private physician, Port Vila, Vanuatu.; h Members of the Vanuatu Ministry of Health’s National Health Emergency Operations Centre are provided in the Acknowledgements.; * These authors contributed equally.

## Abstract

The Pacific island nation of Vanuatu is vulnerable to emerging infectious diseases, including epidemics and pandemics; chronic food and water insecurity; and natural hazards, including cyclones, earthquakes, tsunamis, landslides and flooding. In March 2020, the World Health Organization characterized the outbreak of novel coronavirus disease 2019 (COVID-19) as a global pandemic. By the end of April 2020, Vanuatu had reported no confirmed cases of COVID-19. Data from several sources are collected in Vanuatu’s COVID-19 surveillance system to provide an overview of the situation, including data from case investigations and management, syndromic surveillance for influenza-like illness, hospital surveillance and laboratory surveillance. Review of data collected from January to the end of April 2020 suggests that there was no sustained increase in influenza-like illness in the community and no confirmed cases were identified. Lessons learnt from the early implementation of surveillance activities, the changing landscape of laboratory testing and pharmaceutical interventions, as well as the global experience, particularly in other Pacific island countries, will inform the refinement of COVID-19 surveillance activities in Vanuatu.

Pacific island countries and territories (PICTs) are marked by expansive geography, relatively small populations and diverse cultures. They are also vulnerable to emerging infectious diseases, including epidemics and pandemics, and to natural disasters, including cyclones, earthquakes and tsunamis. For these reasons, the World Health Organization’s *Asia Pacific Strategy for Emerging Diseases and Public Health Emergencies* (APSED III) guides Member States to adopt an all-hazards approach, encompassing both disease outbreaks and natural disasters, to strengthen their capacity to detect, prepare for and respond to outbreaks of infectious diseases and public health emergencies. (*1*)

On 30 January 2020, the WHO Director-General declared that the outbreak of novel coronavirus disease 2019 (COVID-19) constituted a public health emergency of international concern. As of 30 April 2020, six PICTs had confirmed cases of COVID-19. (*2*) In Vanuatu, a country of approximately 290 000 people and composed of 83 islands, the response to COVID-19 is guided by the VanGov Plan (COVID-19 Health Sector Preparedness and Response Plan) developed in January 2020 and revised as the situation evolves. (*3*) Priority actions are categorized according to three scenarios: 1 (no cases), 2 (one or more cases or clusters) and 3 (community transmission). A strategic objective of the plan is to ensure that the surveillance system is active and functional. Since January 2020, the Government of Vanuatu has implemented several measures to prevent the importation of COVID-19 and contain and mitigate community transmission, including suspending the use of international ports of entry into Vanuatu on 23 March 2020 and declaring a state of emergency on 26 March 2020.

We describe the implementation of the initial COVID-19 surveillance system established in Vanuatu between January and April 2020, focusing on its design, challenges and the modifications required.

## Ethics statement

The Vanuatu Health Research Ethics Committee advised that ethics approval was not required because data were being collected as part of the pandemic response and in line with the Vanuatu Public Health Act No. 22 of 1994.

## The surveillance system, modifications and interventions

The objective of the COVID-19 surveillance system in Vanuatu is to rapidly identify and contain any imported or community-acquired cases of COVID-19 (**Table 1**). The framework for surveillance systems suggested by Heymann (*4*) was used to describe the system, which collates data from several sources.

**Table 1:** Main objectives and interventions of the surveillance response to the COVID-19 pandemic, as per the VanGov Plan (COVID-19 Health Sector Preparedness and Response Plan), Vanuatu, January–April 2020

Objectives	Scenario and interventions		
	1 (no cases)	2 ((*3*) 1 case, imported or locally detected [sporadic], OR clusters of cases)	3 (community transmission)
**Early detection and isolation of suspected COVID-19 cases by an active and functional surveillance system**	**Use WHO definition to test suspected cases.** **Train workers at sentinel sites, health-care workers and private practitioners about case definition and notification and reporting channels.**	**Use WHO definition to test suspected cases.** **Provide refresher training to workers at sentinel sites, health-care workers and private practitioners about case definition and notification and reporting channels.** **Enhance syndromic surveillance system, focusing on influenza-like illness and COVID-19 in public health facilities, and enhance event-based surveillance system in private health facilities.** **Test if patient has symptoms, and implement contact tracing and monitoring.**	**Enhance syndromic surveillance system, focusing on influenza-like illness and COVID-19 in public health facilities, and enhance event-based surveillance system in private health facilities.** **Implement sampling strategy for testing, depending on number of suspected cases.**

COVID-19: coronavirus disease 2019; WHO: World Health Organization.

### Existing data collection systems

The Vanuatu Public Health Sentinel Surveillance Network is part of the regional Pacific Public Health Surveillance Network. (*5*) Eleven sites in Vanuatu report weekly on five core syndromes: (i) acute fever and rash, (ii) prolonged fever, (iii) influenza-like illness (ILI), (iv) watery diarrhoea, and (v) illnesses that are like dengue, Zika or Chikungunya. (*5*) These syndromes are monitored as part of the all-hazards approach to tracking infectious diseases related to both outbreaks and natural disasters. Data are compiled weekly and sent to the national surveillance unit via e-mail, phone or short message service (that is, SMS or text), and they are manually entered into a custom Excel database. ILI data are monitored because the symptoms of COVID-19 are clinically similar to influenza (**Table 2**). A pre-established threshold was set (*n* = 426 per week) to generate an alert and prompt action if the number of reported cases is greater than expected for seasonal influenza. Standard reporting is by epidemiological week (epi week), with week 1 ending 5 January 2020.

**Table 2:** Summary of sentinel and hospital surveillance activities related to the COVID-19 pandemic, Vanuatu, January–April 2020

Network or site	Number of sites	Coverage area	Site type (number)	Start date	Type of data used for COVID-19 surveillance
**Vanuatu Public Health Sentinel Surveillance Network**	**11**	**National**	**Hospital (*n* = 6)** **Health centre (*n* = 5)**	**Predated COVID-19**	**ILI**
**General practitioner sentinel sites**	**7**	**Port Vila only**	**Private clinic (*n* = 5)**	**23 March 2020**	**ILI**
**Hospital-based surveillance**	**6**	**National**	**Hospital (*n* = 6)**	**20 March 2020**	**ILI (captured through the Vanuatu Public Health Sentinel Surveillance Network),** **SARI, pneumonia,** **deaths,** **number of paracetamol tablets dispensed**

COVID-19: coronavirus disease 2019; ILI: influenza-like illness; SARI: severe acute respiratory infection.

### Enhancement of systems for COVID-19 surveillance

A sentinel surveillance system for private clinics in Port Vila was established in March 2020 among general practitioners. The objective was to rapidly identify imported cases and monitor community-level transmission of COVID-19 among expatriates, who predominantly use private clinics. Clinics were requested to submit daily reports via a web form of the number of consultations and the number of people presenting with ILI (**Table 2**).

Active hospital-based surveillance activities were established in April 2020 to monitor and rapidly identify any cases of severe acute respiratory infection (SARI) or pneumonia-related presentations to emergency departments, and hospitalizations and deaths. Data were collected daily from the main referral hospital in Port Vila and five provincial hospitals (**Table 2**). In addition, data on the number of tablets of paracetamol dispensed through the emergency department were collected weekly. A surveillance officer contacted all hospitals daily to verbally collect information on new admissions for SARI or pneumonia, and weekly for paracetamol dispensing.

### Case investigation and management

Protocols were developed to investigate all suspected cases: a public health officer interviews all suspected cases to determine whether the person meets the case definition and the possible source of transmission, to identify close contacts and to implement steps to minimize ongoing transmission.

The initial protocol implemented in January 2020 was for suspected cases to be immediately isolated at home to prevent onward transmission; it has since been temporarily revised to implement hospital-based isolation of suspected cases in a specific ward. Hospitalization of suspected cases became necessary due to the length of time required to receive laboratory results (average: 4.1 days) and the need to control the risk of potential transmission during this time.

### Laboratory testing

Vanuatu’s strategy for COVID-19 laboratory testing during the period of interest was to collect and refer for testing specimens from individuals who met WHO’s definition of a suspected case. (*6*) In limited circumstances and in consideration of the global shortage of molecular testing reagents for COVID-19, (*7*) precautionary testing was undertaken for selected additional individuals.

### Isolation and treatment of cases

Since February 2020, the Vanuatu health ministry has undertaken significant measures to strengthen the country’s medical capacity to manage patients with severe COVID-19, including establishing a dedicated intensive care unit for patients needing critical care and a ward for patients with mild disease who cannot isolate at home.

### Contact management, identification, case finding and quarantine

Protocols using WHO’s definition of a close contact (*6*) were established for contact tracing to rapidly identify contacts of confirmed cases to determine possible sources of infection and to prevent onward transmission. The protocol specified that asymptomatic close contacts of confirmed cases were to be quarantined in a designated facility or at home for 14 days from their last date of exposure, as per Section 12 of the Vanuatu Public Health Act No. 22 of 1994, which allows for the isolation and detainment of a person recently exposed to infection or who may be in the incubation stage of any notifiable disease. (*8*) If close contacts developed symptoms, as per the WHO case definition, (*6*) they were to be referred to hospital for isolation and testing.

### Management of international arrivals

Quarantine in a government-designated facility for a period of 14 days is required for all people arriving in Vanuatu from 20 March 2020 onwards. Protocols were developed to monitor people in quarantine: provincial public health teams conducted daily visits to screen for symptoms of respiratory illness and fever. All people working in the quarantine facilities, including transport providers, hotel front desk clerks, cleaners, kitchen workers and security officers received training from the Vanuatu Ministry of Health.

## Implementation of the new system January–April 2020

### Existing systems

The number of ILI cases reported through the Vanuatu Public Health Sentinel Surveillance Network fluctuated between epi week 1 (EW1) and EW18 (range: 156–489; **Table 3**). In EW18, there were 212 reports of ILI, a decrease of 25 from the previous week (*n* = 237). The number of ILI reports did not reach the threshold during the period (**Table 3**).

**Table 3:** Data collected through various surveillance activities for COVID-19, by epidemiological week (epi week), Vanuatu, January–April 2020

Week		-	Indicator (system)			
Start date	End date	Epi week	Influenza-like illness (Vanuatu Public Health Sentinel Surveillance Network)	Influenza-like illness (private clinic syndromic surveillance)	Pneumonia (hospital surveillance)	Number of tablets of paracetamol dispensed through emergency department
**30/12/2019**	**5/01/2020**	**1**	**489**	**NC**	**NC**	**NC**
**6/01/2020**	**12/01/2020**	**2**	**250**	**NC**	**NC**	**NC**
**13/01/2020**	**19/01/2020**	**3**	**205**	**NC**	**NC**	**NC**
**20/01/2020**	**26/01/2020**	**4**	**341**	**NC**	**NC**	**NC**
**27/01/2020**	**2/02/2020**	**5**	**191**	**NC**	**NC**	**NC**
**3/02/2020**	**9/02/2020**	**6**	**238**	**NC**	**NC**	**NC**
**10/02/2020**	**16/02/2020**	**7**	**205**	**NC**	**NC**	**NC**
**17/02/2020**	**23/02/2020**	**8**	**171**	**NC**	**NC**	**NC**
**24/02/2020**	**1/03/2020**	**9**	**319**	**NC**	**NC**	**NC**
**2/03/2020**	**8/03/2020**	**10**	**198**	**NC**	**NC**	**NC**
**9/03/2020**	**15/03/2020**	**11**	**292**	**NC**	**NC**	**NC**
**16/03/2020**	**22/03/2020**	**12**	**273**	**NC**	**NC**	**NC**
**23/03/2020**	**29/03/2020**	**13**	**268**	**18**	**NC**	**NC**
**30/03/2020**	**5/04/2020**	**14**	**224**	**45**	**4**	**50**
**6/04/2020**	**12/04/2020**	**15**	**156**	**40**	**4**	**170**
**13/04/2020**	**19/04/2020**	**16**	**209**	**14**	**2**	**915**
**20/04/2020**	**26/04/2020**	**17**	**237**	**6**	**1**	**1340**
**27/04/2020**	**3/05/2020**	**18**	**212**	**13**	**1**	**790**

NC: data not collected before March 2020 when additional surveillance activities were implemented.

### Enhancement for COVID-19 surveillance

Among reports submitted from seven private clinics in the general practitioners’ sentinel surveillance system between EW14 and EW18, there were also fluctuations in the number of consultations for ILI (range: 6–45), and a sustained increase was not observed (**Table 3**).

Only pneumonia-related hospitalization data were available for the period; SARI data were not available. Pneumonia hospitalization data were received from five of six hospitals in Vanuatu beginning in EW14. The number of new admissions for pneumonia decreased from four to one between EW14 and EW18 (**Table 3**). The number of paracetamol tablets dispensed through the emergency department was greatest in EW17 (*n* = 1340, **Table 3**).

### Enhancing case investigation and management

Between January and April 2020, two people met the WHO case definition of a suspected case. Both patients had symptoms of ILI and had recently travelled overseas. Both of these patients isolated at home until the results of their COVID-19 tests were known. These patients were reported as suspected cases on 19 March and 30 March 2020.

### Laboratory testing of specimens

Between January and April 2020, COVID-19 testing was not available in Vanuatu, and all specimens were sent to New Caledonia for molecular testing. As of 30 April 2020, 24 specimens from 19 people had been sent to New Caledonia; of these, specimens were from eight people identified in private clinics (42%), two people from government-run health clinics (11%) and the remainder (*n* = 9; 47%) were identified through the Vila Central Hospital emergency department or outpatient clinic. Due to border control measures, each dispatch of samples required government approval and significant logistical coordination. The average number of days from specimen collection to test result was 4.1, with a range of 1–12 days. The samples from the two patients who met the WHO definition of a suspected case had test results in 2 and 5 days and both were identified by private clinics. The remainder of cases did not meet the WHO case definition and so had precautionary tests. None of the samples tested during this period was positive.

### Isolation and treatment

As there were no confirmed cases during the study period, the isolation and treatment of cases was not required.

### Contact management and quarantine

As there were no confirmed cases during the study period, contact tracing was not initiated.

### Managing international arrivals

As of 30 April 2020, a total of 98 people arriving from overseas had completed quarantine. The majority (*n* = 61; 62%) were passengers on the two last flights arriving into Vanuatu on 21 March 2020 before the border was closed.

## Discussion

The aims of a national surveillance system depend on a country’s pandemic response strategy as well as the local epidemiological context and laboratory and health facility capacities. The objectives may be to identify severe cases, asymptomatic cases, clusters of cases or a combination of these. Because no cases have been detected in Vanuatu as of 30 April 2020, the aims of surveillance for COVID-19 are to rapidly detect and contain any imported cases. Achieving these aims relies on timely and accurate laboratory testing. The absence of in-country testing between January and April 2020 significantly limited Vanuatu’s initial capacity to respond effectively to the COVID-19 threat.

For most PICTs, including Vanuatu, in-country laboratory testing was not available until May 2020. If a case had been detected before May, the capacity of the country to implement timely containment and mitigation measures would have been reduced due to the lag between specimen collection and receiving results. In March 2020, a rapid molecular test using the  GeneXpert platform (Cepheid, Sunnyvale, CA, USA), which provides fully automated, easy-to-use point-of-care molecular testing, (*9*) was approved for COVID-19 testing by the US Food and Drug Administration. The Joint Incident Management Team (coordinated by the WHO Representative Office in the South Pacific) procured GeneXpert cartridges and machines from the manufacturer for distribution across PICTs. (*10*) As a result, in-country laboratory testing in Vanuatu became available in May 2020, and this has strengthened Vanuatu’s capacity to respond to COVID-19. A testing strategy has been developed that considers both the epidemiological situation in Vanuatu and the anticipated limited availability of cartridges due to staggered distribution and the global shortage of consumables, including swabs.

The absence of confirmed cases in Vanuatu and elsewhere cannot be interpreted as an absence of circulating virus, especially in countries where there is limited testing capacity. Currently, there is no international guidance about how to verify the absence of circulating virus. Data collected by the various syndromic surveillance systems in Vanuatu will continue to be used to monitor and verify the absence of confirmed cases. Internationally, severe and critical cases comprise around 20% of diagnosed cases of COVID-19 (*11*) and, therefore, we assume that any undetected circulating virus would result in an increase in ILI in primary health care facilities and pneumonia in hospitals.

In the context of having no confirmed cases and in the absence of widespread availability of pharmaceutical interventions, such as treatment or vaccination, reopening the border may result in the importation of COVID-19 to Vanuatu. The various surveillance components described here are critical to rapidly detecting and containing any imported cases. Mathematical modelling data are not available to enable Vanuatu to predict the impact of imported cases using current population data and COVID-19 parameters, but they would be useful to guide the evolving response.

Several PICTs were also affected by Tropical Cyclone Harold in April 2020. (*12*) Harold impacted Vanuatu on 6–7 April 2020 as a category 5 cyclone. More than 160 000 people, approximately 55% of the population, reside in areas that were affected by the cyclone. (*13*) Harold occurred during a period of rapid scale-up and strengthening of COVID-19 surveillance activities. The implementation and strengthening of ILI surveillance in provinces affected by the cyclone were complicated by the emergence of several post-disaster outbreak-prone diseases that also have symptoms of ILI, such as dengue and leptospirosis. Where possible, the Vanuatu health ministry sought to harmonize surveillance activities, as demonstrated through the collection of data about ILI and injuries through pre-existing and new surveillance activities. Strategies to conduct disease surveillance for two events simultaneously at such a large scale is unprecedented in Vanuatu and elsewhere, and guideline developers should consider providing information about how to respond to a similar situation in the future.

Several additional limitations should be considered when assessing the implementation of Vanuatu’s COVID-19 surveillance; these include pre-existing shortages of clinical and public health workers, limited pre-existing epidemiological capacity within Vanuatu’s health ministry, the country’s geographical isolation and small population, and its limited laboratory capacity. Nonetheless, the Vanuatu health ministry and its partners have rapidly scaled up surveillance activities in a complex, challenging and rapidly changing epidemiological landscape.

The COVID-19 response is continuing in Vanuatu and will adapt as the epidemiological context changes. Lessons from the early implementation of surveillance activities during Scenario 1 (no cases), the changing landscape of laboratory testing and pharmaceutical interventions, as well as the global experience, particularly in other PICTs, will inform the refinement of COVID-19 surveillance activities in Vanuatu.
